# Supplementary material for: Inhibition of Staphylococcus aureus LC 554891 by Moringa oleifera Seed Extract either Singly or in Combination with Antibiotics
Source: Molecules. 2020 Oct 7;25(19):4583. doi: 10.3390/molecules25194583 (PMC7582841; doi:10.3390/molecules25194583)
Supplement: Supplementary file 1 [file molecules-25-04583-s001.pdf]

Supplementary Material

Supplementary Table 1. Incidence of presumptive *Staphylococcus aureus* bacteria in different samples of beef luncheon, chips and corn flakes.

Type of food sample	Total number of samples tested	Number of samples positive for SAC	% Incidence	No. and % of CFU/g> 5 $\times 10^3$ counts
Beef luncheon	25	11	44	5^+(45.45%)*
Chips	50	7	14	2^+ (28.57%)*
Corn flakes	25	3	12	0

+ Number of food samples showed *S. aureus* counts > 5×10^3 CFU/g. * % of samples showed SAC counts >5×10^3CFU/g within all the positive samples for SAC

Supplementary Table 2. Identification of 30 presumptive *S. aureus* isolates by the biochemical reactions by fermentation of different sugars via API system:

Code of Isoltes	API tests																				Name of isolates
	0	GLU	FRU	MN	MA	LAC	TRE	MA	XLT	ME	NIT	PAL	VP	RAF	XYL	SAC	MD	NA	AD	URE	
B3	-	+	+	+	+	+	+	+	-	-	+	+	+	-	-	+	-	+	+	-	*S. aureus(B3)*
B7	-	+	+	+	+	+	+	+	-	-	+	+	+	-	-	+	-	+	+	-	*S. aureus(B7)*
B8	-	+	+	+	+	+	+	+	-	-	+	+	+	-	-	+	-	+	+	-	*S. aureus(B8)*
B14	-	+	+	+	+	+	+	+	-	-	+	+	+	-	-	+	-	+	+	-	*S. aureus(B14)*
B17	-	+	+	+	+	+	+	+	-	-	+	+	+	-	-	+	-	+	+	-	*S. aureus(B17)*
B18	-	+	+	+	+	+	+	+	-	-	+	+	+	-	-	+	-	+	+	-	*S. aureus(B18)*
B22	-	+	+	+	+	+	+	+	-	-	+	+	+	-	-	+	-	+	+	-	*S. aureus(B22)*
B24	-	+	+	+	+	+	+	+	-	-	+	+	+	-	-	+	-	+	+	-	*S. aureus(B24)*
Ch32	-	+	+	+	+	+	+	+	-	-	+	+	+	-	-	+	-	+	+	-	*S. aureus(Ch32)*
Ch35	-	+	+	+	+	+	+	+	-	-	+	+	+	-	-	+	-	+	+	-	*S. aureus(Ch35)*
Ch40	-	+	+	+	+	+	+	+	-	-	+	+	+	-	-	+	-	+	+	-	*S. aureus(Ch40)*
Ch41	-	+	+	+	+	+	+	+	-	-	+	+	+	-	-	+	-	+	+	-	*S. aureus(Ch41)*
Ch48	-	+	+	+	+	+	+	+	-	-	+	+	+	-	-	+	-	+	+	-	*S. aureus(Ch48)*
Ch50	-	+	+	+	+	+	+	+	-	-	+	+	+	-	-	+	-	+	+	-	*S. aureus(Ch50)*
Ch53	-	+	+	+	+	+	+	+	-	-	+	+	+	-	-	+	-	+	+	-	*S. aureus(Ch53)*
Cf58	-	+	+	+	+	+	+	+	-	-	+	+	+	-	-	+	-	+	+	-	*S. aureus(Cf58)*
Cf66	-	+	+	+	+	+	+	+	-	-	+	+	+	-	-	+	-	+	+	-	*S. aureus(Cf66)*
Cf69	-	+	+	+	+	+	+	+	-	-	+	+	+	-	-	+	-	+	+	-	*S. aureus(Cf69)*

Enzyme activities (19 enzymes) were measured using the commercial API® ID 32 STAPH including: GLU (D-glucose), FRU (D-fructose), MNE (D-mannose), MAL (D-maltose), LAC (D-lactose), TRE (D-tréhalose), MAN (D-mannitol), XLT (xylitol), MEL (D-mélibiose), NIT (nitrate de potassium), PAL (ß-naphtyl phosphate), VP (sodiumpyruvate), RAF (D-raffinose), XYL (D-xylose), SAC (D-saccharose), MDG (méthyl-DDglucopyranoside), NAG (N-acétyl-glucosamine), ADH (L-arginine), URE (urée). Symbols B; Ch; Cf mean that the isolate obtained from beef; potato chips; corn flaxes respectively.

ATTACTAGCGATTCCAGCTTCATGTAGTCGAGTTGCAGACTACAATCCGAACTGAGA
ACAACTTTATGGGATTTGCTTGACCTCGCGGTTTCGCTGCCCTTTGTATTGTCCATTG
TAGCCGTGTGTAGCCCAAATCATAAGGGGCATGATGATTTGACGTCATCCCCACCTT
CCTCCGGTTTGTCACCGGCAGTCAACTTAGAGTGCCCAACTTAATGATGGCAACTAA
CTTAAGGGTTGCGCTCGTTGCGGGACTTAACCCAACATCTCACGACACGAGCTGACG
ACAACCATGCACCACCTGTCACTTTGTCCCCCGAAGGGGAAAGCTCTATCTCTAGAG
TTGTCAAAGGATGTCAAGATTTGGTAAGGTTCTTGCGTTGCTTCGAATTAAACCACA
TGCTCCACCGCTTGTGCGGGCCCCGTCAATTCCTTTGAGTTTCAACCTTGCGGTCGTA
CTCCCCAGGCGGAGTGCTTAATGCGTTAGCTGCAGCACTAAGGGGCGGAAACCCTA
ACACTTAGCACTCATCGTTTACGGCGTGGACTACCAGGGTATCTAATCCTGTTTGAT
CCCCACGCTTTCGCACATCAGCGTCAGTTACAGACCAGAAAGTCGCCTTCGCCACTG
GTGTTCCTCCATATCTCTGCGCATTTCACCGCTACACATAATTCCACTTTCCTCTTCTG
CACTCAAGTTTTCCAGTTTCCAATGACCCTCCACGGTTGAGCCGTGGGCTTTCACATC
AGACTTAAAAAACCGCCTACGCGCGCTTTACGCCCAATAATTCCGGATAACGCTTGC
CACCTACGTATTACCGCGGCTGCTGGCACGTAGTTAGCGTGGCTTTCTGATTAGGTA
CCGGTCAGATGTGCACAGTTACTTACACATATGTTCTTCCTAATAACAGAAGTTTAC
GATCCGAGAACATC

Supplementary Figure 1. Nucleotide sequence of 16S r RNA gene of *S. aureus* B3;

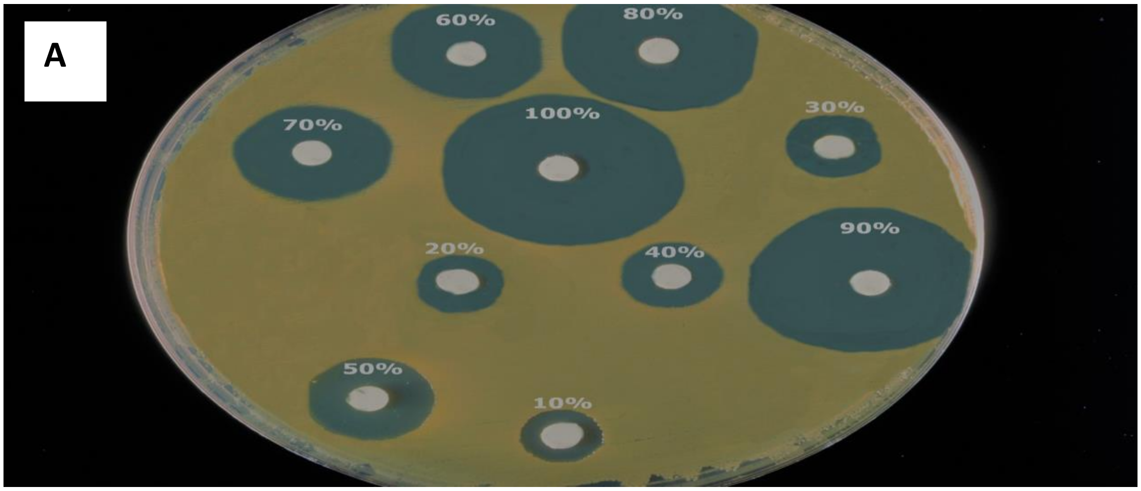

Supplementary Figure 2A. Antibacterial activity of crude honey using disc diffusion assay was shown at (10 %) cause inhibition zone (13mm) against *S. aureus* (B3).

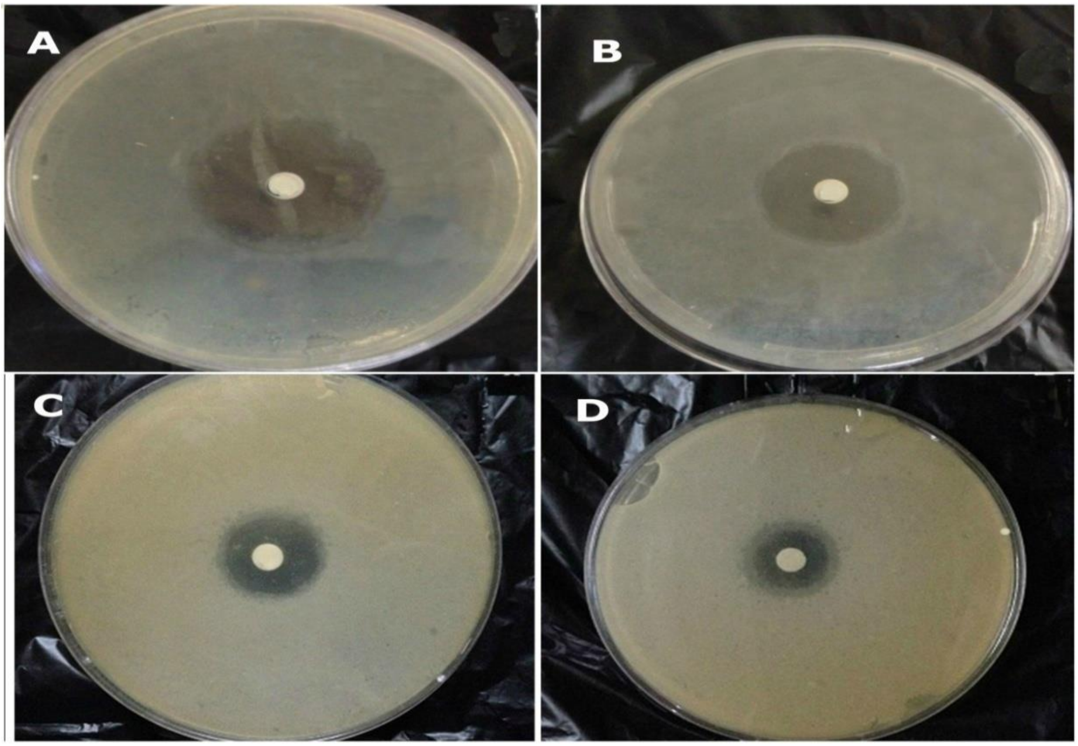

Supplementary Figure 2B. Antibacterial activity of the initial aqueous extract (10µg/100mL water) of either MSE against *S. aureus* LC 554891 (A) and *S. aureus* ATCC6538 (C) or MLE against *S. aureus* LC 554891 (B) and *S. aureus* ATCC6538 (D) by disc diffusion assay.

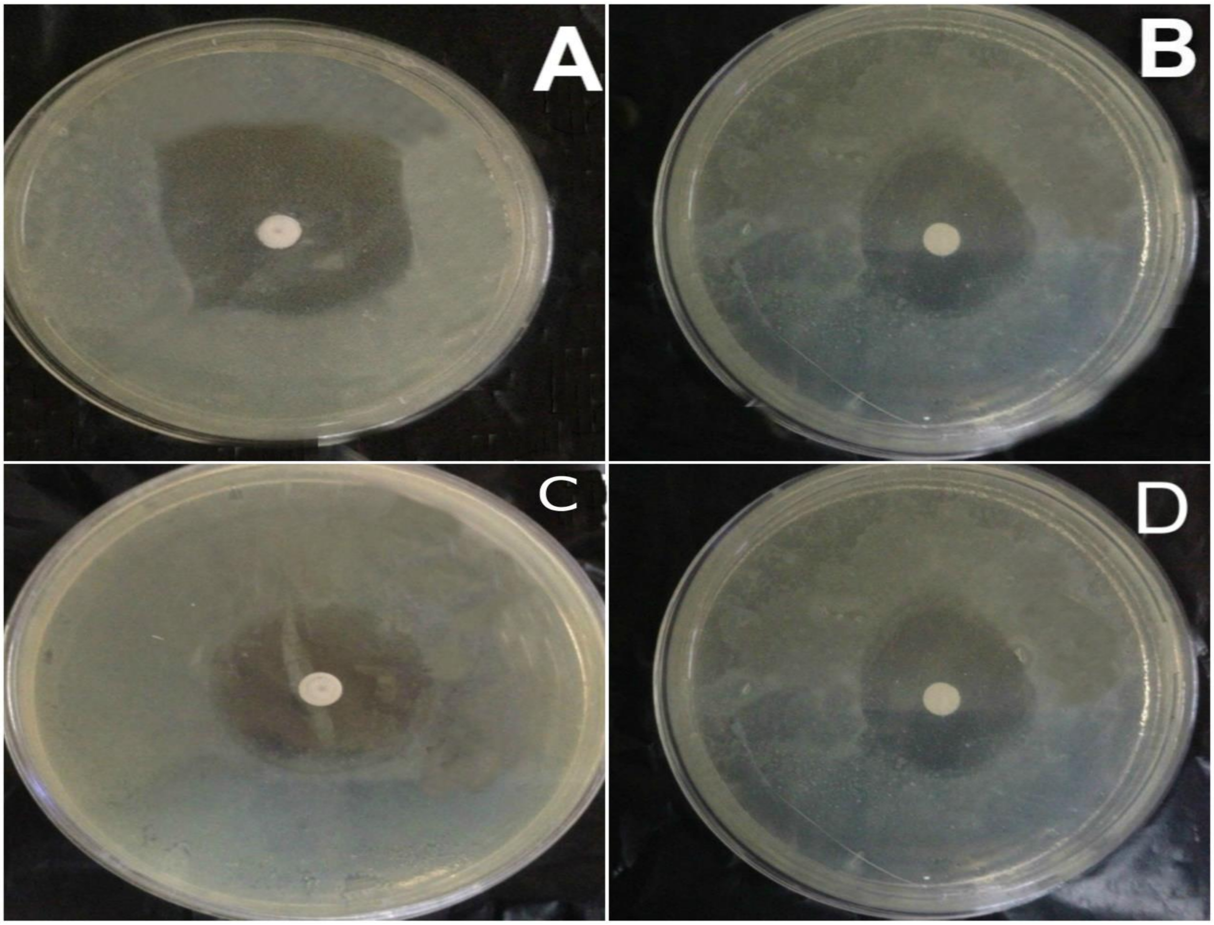

Supplementary Figure 3. Antibacterial activity of (A) & (B): Moringa oil with inhibition zone (37 & 35 mm) at conc. (0.5%) against *S. aureus* (ATCC 6538) and *S. aureus* LC 554891, respectively, (C) &(D): Moringa oil with inhibition zone (35 & 30 mm) at conc. (0.25%) against *S. aureus (ATCC 6538) and S. aureus* LC 554891 respectively by disc diffusion method.
